# Research on the Erosion Law and Protective Measures of L360N Steel for Surface Pipelines Used in Shale Gas Extraction

**DOI:** 10.3390/ma17174278

**Published:** 2024-08-29

**Authors:** Shaoquan Huo, Lincai Peng, Yunpeng Li, Yong Xu, Hongbing Huang, Xi Yuan

**Affiliations:** 1 Research Institute of Natural Gas Technology, PetroChina Southwest Oil and Gasfield Company, Chengdu 610213, China; 2 Chengdu Natural Gas Chemical Plant, PetroChina Southwest Oil and Gasfield Company, Chengdu 610213, China; 3 PetroChina Southwest Oil and Gasfield Company, Chengdu 610051, China

**Keywords:** shale gas, surface pipeline, L360N, erosion, surface protection

## Abstract

The erosion of surface pipelines induced by proppant flowback during shale gas production is significant. The surface pipelines in a shale gas field in the Sichuan Basin experienced perforation failures after only five months of service. To investigate the erosion features of L360N, coatings, and ceramics and optimize the selection of two protective materials, a gas–solid two-phase flow jet erosion experimental device was used to explore the erosion resistance of L360N, coatings, and ceramics under different impact velocities (15 m/s, 20 m/s, and 30 m/s). An energy dispersive spectroscope, a scanning electron microscope, and a laser confocal microscope were employed to analyze erosion morphologies. With the increase in flow velocity, the erosion depth and erosion rate of L360N, coating, and ceramic increased and peaked under an impact velocity of 30 m/s. The maximum erosion rate and maximum erosion depth of L360N were, respectively, 0.0350 mg/g and 37.5365 µm. Its primary material removal mechanism was the plowing of solid particles, and microcracks were distributed on the material surface under high flow velocities. The maximum erosion rate and maximum erosion depth of the coating were, respectively, 0.0217 mg/g and 18.9964 µm. The detachment of matrix caused by plowing is the main material removal mechanism. The maximum erosion rate and maximum erosion depth of ceramics were, respectively, 0.0108 mg/g and 12.4856 µm. The erosion mechanisms were micro-cutting and plowing. Under different particle impact velocities, different erosion morphologies were observed, but the primary erosion mechanism was the same. The erosion resistance of the ceramics was higher than that of the coatings. Therefore, ceramic lining materials could be used to protect the easily eroded parts, such as pipeline bends and tees, and reduce the failure rate by more than 93%. The study provided the data and theoretical basis for the theoretical study on oil and gas pipeline erosion and pipeline material selection.

## 1. Introduction

Shale gas resources in China rank first in the world, and the total reserves are more than 2000 trillion m^3^ and widely distributed in the Sichuan Basin, the Ordos Basin, and other regions [1]. The large-scale hydraulic fracturing technology is applied in the development of shale gas and has improved mining efficiency and gas production, but it has also brought out a large number of problems caused by proppant flowback [1,2,3]. In the exploitation and transportation process of shale gas, after desanding, the high-viscosity and high-stability fracturing flowback fluid still contains about 10% of sand particles before entering pipelines, thus causing pipeline erosion and wear [4,5], which seriously affect the flowback efficiency and the safety of shale gas exploitation.

The erosion process of shale gas pipelines under a gas–solid two-phase flow or a multi-phase flow is complex. Such flows are more prone to lead to sudden changes in flow patterns at some locations, such as elbows, tee joints, and valves. The instantaneous impact velocity of the fluid is large, and solid particles repeatedly collide with the inner wall of pipelines, thus leading to safety incidents such as wall thinning, perforation, and even damage and leakage of pipelines [6,7]. The erosion of surface pipelines in shale gas fields is affected by many factors, including the characteristics of the target material, shape, particle size, hardness, impact velocity, impact angle, and flow characteristics [5,6,8,9,10,11,12]. The erosion mechanism of shale gas pipelines has been extensively explored at home and abroad. Liu et al. [11] analyzed the erosion mechanism of elbows in the Changning Shale Gas Field under different impact angles and indicated that the erosion rate and erosion area of elbows were mainly influenced by gas flow velocity and particle aggregation based on FLUENT simulation. With a two-way coupled Euler–Lagrange method in computational fluid dynamics (CFD) and response surface methodology (RSM), Chi et al. [4] investigated the factors of elbow erosion in shale gas gathering and transportation systems and found that the maximum erosion rate was positively correlated with bending angle, bending direction, particle size, particle velocity, and particle mass flow rate and negatively correlated with the inner diameter and R/D ratio of pipelines. Furthermore, the maximum erosion rate was influenced by the interaction of any two factors. Jia et al. [13] constructed a numerical model in FLUENT to explore the synergistic process of erosion and corrosion of 90° elbows in shale gas pipelines under gas–liquid–solid multi-phase flow conditions. Zhu et al. [14] studied the effects of inlet velocity, curvature diameter ratio, and pipe diameter on the erosion rate of 90° elbows through numerical simulations based on FSI-CFD and DPM. The previous studies on the erosion induced by proppant flowback during the production process of oil and gas wells mainly focused on numerical simulation [15,16,17]. However, experimental studies on gas–solid erosion damage in shale gas gathering and transportation pipelines or the influences of various environmental factors on pipeline material erosion damage were seldom reported.

Proppant flowback caused by large-scale hydraulic fracturing technology poses a serious erosion threat to pipelines. Therefore, it is significant to select appropriate protective materials and optimize material surface properties in the long-term service of shale gas pipelines. To address the problem of erosion in surface pipelines induced by proppants in shale gas gathering and transportation systems, surface treatment technologies such as electroplating, anodic oxidation, and ion implantation are increasingly applied in the protection of pipelines and equipment [18,19]. In recent years, high-hardness materials such as tungsten carbide, cemented carbide, and ceramics have attracted wide attention from the fields of materials science and petroleum engineering. These materials possess significant advantages in hardness, wear resistance, high-temperature resistance, and corrosion resistance for pipeline protection [20,21,22,23,24,25,26].

Surface pipelines in a shale gas field in the Sichuan Basin were made of L360N steel. After five months of service, one perforation occurred at the elbows of drainage pipelines, and another perforation emerged three days after repair welding. The eroded hole was 4 cm long and 2 cm wide. The connecting pipe from the wellhead to the desanding skid failed after two months of service, and the erosion depth was 3 to 4 mm. A metal coating or ceramics should be selected as protective materials for pipelines. Taking L360N steel, coatings, and ceramics as research objects, a gas–solid two-phase flow jet erosion experiment was conducted with a high-speed gas–sand jet erosion tester in order to explore the erosion patterns and mechanisms of L360N steel, coatings, and ceramics under different impact velocities, at an impact angle of 30° in the study. Based on the experimental results, the protective material was selected from coatings and ceramics. The study provided the data and theoretical basis for the erosion theory research, engineering applications, and material selection of oil and gas pipelines.

## 2. Materials and Methods

### 2.1. Materials

**Figure 1:**
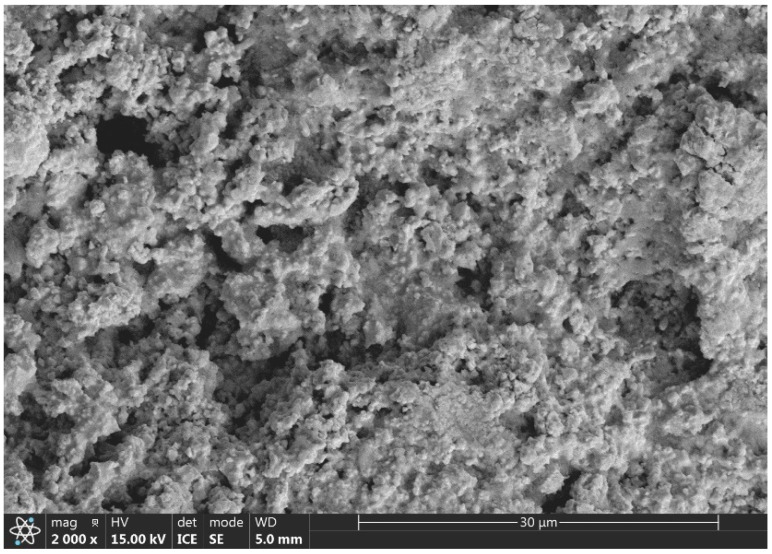
Microstructure of the coating.

The materials used in the experiment were L360N, ceramics, and coatings. According to the ASTM-G76 standard [27], the materials were cut into specimens with dimensions of 20 × 20 × 5 mm. A spark-source atomic emission spectrometer was used to analyze the chemical composition of the three materials (Table 1, Table 2 and Table 3). The L360N steel tube undergoes a normalizing heat treatment process before use, which significantly enhances the material’s strength and toughness, ensuring it meets the mechanical requirements of pipeline transportation. The ceramic material used is ZrO_2_ ceramic (ZrO_2_, 94.2%, sourced from Beijing Jinkelong Petroleum Technology Development Co., Ltd., Beijing, China). This ceramic is prepared using yttria-stabilized zirconia powder as the experimental raw material. A chemical co-precipitation method is employed to produce high-purity, uniformly dispersed powder. The powder is then pressed into a preform of the desired shape using cold isostatic pressing technology. Following this, the preform is sintered at 1600 °C for 3 h under pressureless conditions, resulting in a dense, high-performance ceramic material. The coating is fabricated through electrodeposition technology by Hunan Nafier New Material Technology Co., Ltd. (Changsha, China) The total thickness of the coating is approximately 50 µm. Before the experiment, the L360N steel undergoes pretreatment such as cleaning and derusting to ensure a clean surface. The electrolyte solution is prepared using industrial-grade pure reagents and deionized water. During the electrodeposition process, a cobalt plate serves as the anode, while L360N steel serves as the cathode. The process takes place at a temperature of 55–70 °C, resulting in a tungsten–cobalt alloy coating with high hardness, wear resistance, and corrosion resistance. The SEM image of the coating is shown in Figure 1.

According to the ASTM-G76 standard, quartz sand was selected as the erosion particle. The particle size of quartz sand was determined with a Master sizer 2000 laser particle size analyzer (Figure S1). The particle size of quartz sand ranged from 20 µm to 126 µm, with an average particle size of 48.479 µm. The size of 90% of the particles were between 20 µm and 80 µm. The above data met the requirement for a median particle size of 50 µm in the ASTM-G76 standard. The microscopic morphology of quartz sand is shown in Figure S2. Macroscopically, quartz sand appeared as a white powder with irregular shapes of different sizes. It had distinct edges and sharp corners and could simulate the erosion and wear of sand particles on pipelines and protective coatings during shale gas extraction, drainage, and production. Before each experiment, quartz sand was dried in an oven at 150 °C for 2 h to ensure that particles in the sand-carrying fluid were in a dry state.

### 2.2. Method

#### 2.2.1. Experimental Device

**Figure 2:**
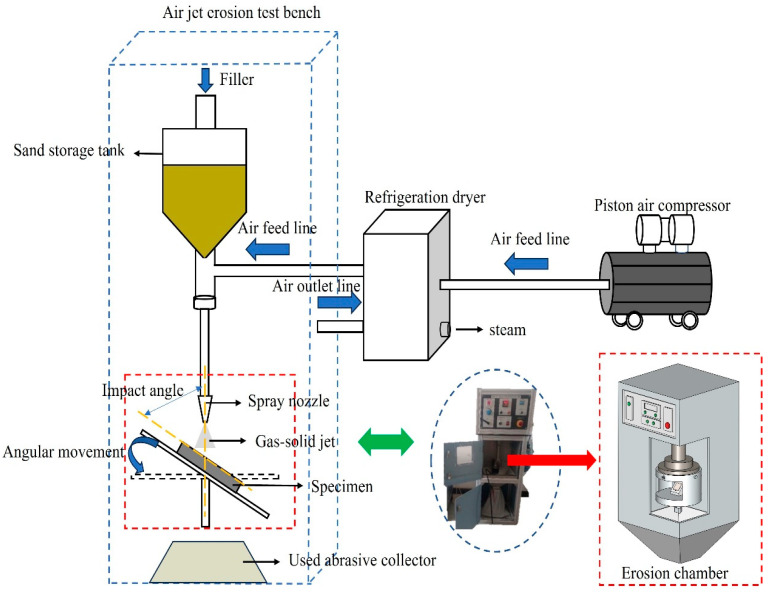
Experimental device for jet erosion tests of a gas–solid two-phase flow.

In this experiment, the AIR JET EROSION TESTER TR-470 (Ducom Instruments, New York, NY, USA) model specified in the ASTM-G76 standard was selected to carry out gas–solid two-phase jet erosion tests on L360N, ceramic, and coating under different flow velocities at normal temperature. As shown in Figure 2 [28], the experimental device is composed of an air jet erosion test bench, a piston air compressor, and a refrigeration dryer. The specimen was placed below the nozzle, and the impact angle of the specimen could be changed through the specimen holder (Figure 2). The distance between the nozzle and the specimen surface was 10 mm, and the nozzle diameter was 1.5 mm. During the solid particle erosion process, air was pressurized in the piston air compressor, and then the moisture in the air flow was removed with the freeze dryer. Then, air was mixed evenly with the particles flowing out of the sand storage tank and accelerated within the reducing tube. Finally, air and particles impacted the target material at a constant speed, thus causing the mass loss of specimen materials.

#### 2.2.2. Measurement Method

**Figure 3:**
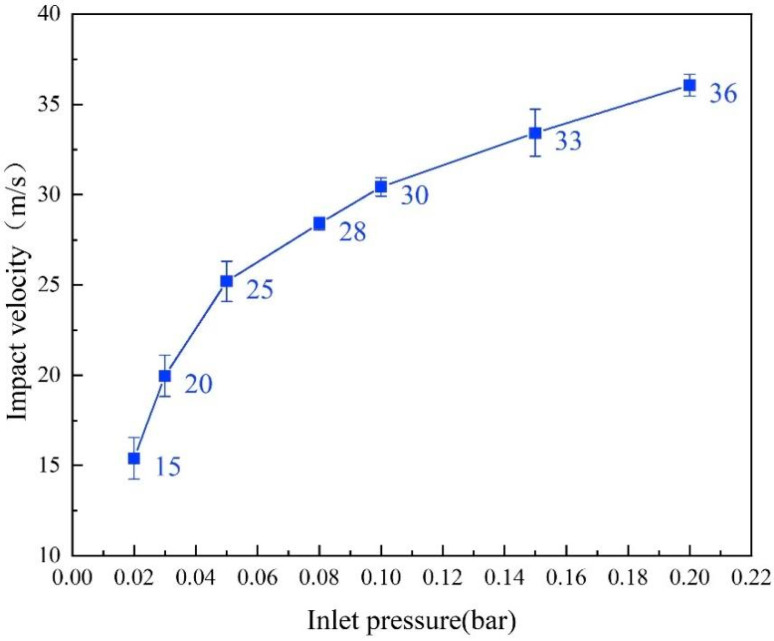
Relationship between inlet pressure and particle impact velocity.

The particle impact velocity was not equivalent to the gas flow velocity, so the double-disk method was adopted to measure the particle impact velocity under different inlet air pressures with a measurement accuracy of ±2 m/s. The particle impact velocity was calculated with Equation (1). The relationship between inlet air pressure and particle impact velocity is shown in Figure 3 [27]. In this experiment, the weight loss method was used to investigate the erosion features of specimens. The erosion rate of specimens was calculated with the ratio of the mass loss of specimens after erosion to the mass of solid particles used during the erosion process (Equation (2)) [29].

(1) $$V=H\times \left(N/60\right)\times \left(360/A\right)$$

where *V* indicates particle impact velocity (m/s); *H* indicates the distance between the upper and lower disks (m); *N* indicates the rotation speed of the motor (krpm); and *A* indicates the mean of the measured angle (°).

(2) $$E=1000\times \left(M_{2}-M_{1}\right)/\left(Q_{m}\times t\right)$$

where *E* indicates erosion rate (mg/g); *M*_2_ indicates the mass of the specimen after testing (g); *M*_1_ indicates the mass of the specimen before testing (g); *Q_m_* indicates mass flow rate of particles (g/min); and *t* indicates erosion time (min).

To reduce experimental errors caused by surface roughness, before testing, the specimens were gradually polished with different waterproof sandpapers (Grit 600, Grit 800, Grit 1000, Grit 1200, and Grit 2000) and then polished. After rinsing with tap water, dehydrating with alcohol, and drying with cold air, the specimens were weighed with an electronic balance (accuracy = 0.1 mg). After the experiment, the residual quartz sand on the specimen surface was removed with anhydrous ethanol to weigh the specimen again and calculate the mass loss of specimens after erosion. The micro-morphology and chemical composition of the eroded area were analyzed with an FEI Quanta450 (FEI, Hillsboro, OR, USA) scanning electron microscope (SEM) and an energy dispersive spectrometer (EDS) at an acceleration voltage of 20 kV. A laser confocal microscope was used to observe the three-dimensional morphology and erosion depth of the eroded area. The local erosion rate was calculated to explore the erosion mechanism.

#### 2.2.3. Experimental Scheme

A gas–solid two-phase flow jet erosion experiment was conducted with L360N, coatings, and ceramics under simulated working conditions with an impact angle of 30° and impact velocities of 15 m/s, 20 m/s, and 30 m/s. The experiment was carried out under a normal temperature and pressure. The erosion time for each specimen was 20 min, and the mass flow rate of particles was 2 g/min. Five parallel specimens were set for each testing condition so as to ensure the accuracy and stability of the experimental results. The experimental parameters are shown in Table 4.

## 3. Results and Discussion

### 3.1. Vickers Hardness Analysis

Before the experiment, the specimens were polished with sandpaper. A Huayin HVS-1000 Vickers (Laizhou Huayin Testing Instrument Co., Ltd., Laizhou, China) hardness tester was used to measure the Vickers hardness of materials. To minimize measurement errors and calculate the average, Vickers hardness was measured at five locations on the specimen surface (Table 5), and the applied load during the experiment was 1000 gf. The average hardness of L360N, ceramics, and coating, respectively, reached 231 HV, 1645 HV, and 1072 HV. Among the three materials, ceramic exhibited the highest hardness, followed by coating, and L360N had the lowest hardness. It is generally believed that the material with the highest hardness has a better erosion resistance [30].

### 3.2. Macroscopic Morphology Analysis

**Figure 4:**
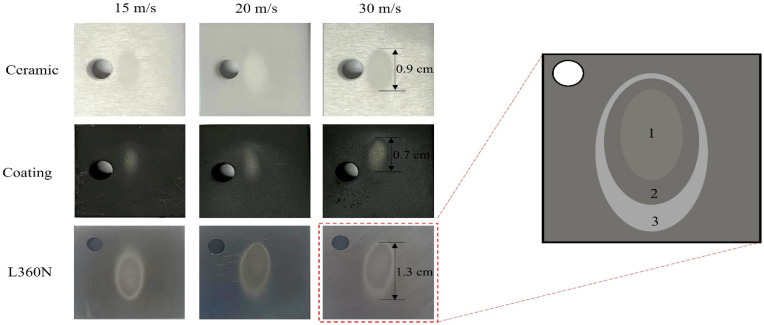
Macroscopic morphologies of L360N, ceramics, and coating under different impact velocities.

The macro-morphologies of L360N, ceramic, and coating under different impact velocities are shown in Figure 4. Under low erosion angles and different flow velocities, the erosion area gradually increased with the increase in impact velocity. The erosion area was the smallest under an impact velocity of 15 m/s and the largest under an impact velocity of 30 m/s. The erosion area showed significant differences among different materials, and the erosion area of L360N was much larger than that of ceramics and coatings. After erosion, the eroded area appeared as a cone with a larger upper part and a smaller lower part, and the cone opening formed an elongated irregular ellipse. According to the degree of erosion damage on the specimen surface, the erosion area could be divided into three parts: the elliptical central part with the highest degree of erosion (Part 1), the elliptical transition part with moderate erosion (Part 2), and the elliptical boundary area with slight erosion and a lighter color (Part 3). In addition, the projected area of the nozzle was smaller than the erosion area (Figure 4). The contour of the upper erosion area of the test piece highly overlapped with the contour of the nozzle projection, but the lower erosion area of the test piece was much larger than the nozzle projection area. The difference might be interpreted as follows: Particles exhibited a higher degree of divergence under the erosion conditions of a small impact angle and were scattered over a larger area on the specimen surface, thus forming elliptical erosion pits during the impact process [31].

### 3.3. Influence of Impact Velocity on Erosion Rate

**Figure 5:**
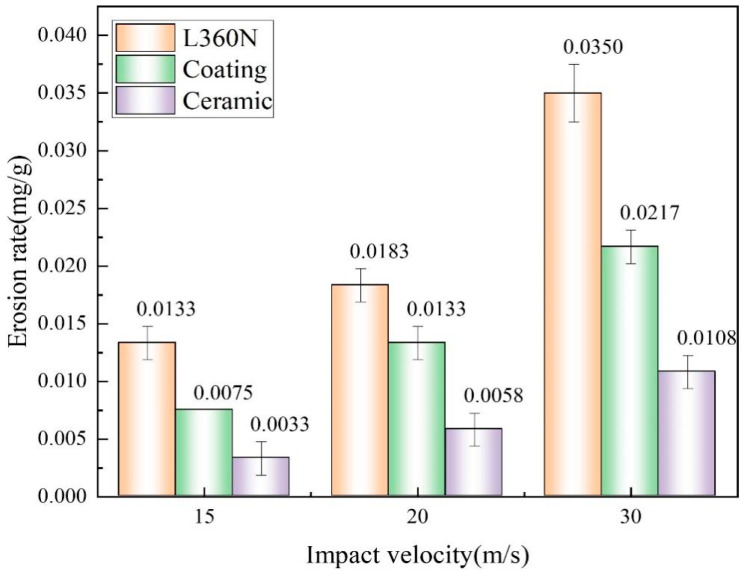
Erosion rates of L360N, ceramics, and coating under different impact velocities.

Figure 5 shows the erosion rates of L360N, ceramic, and coating after erosion for 20 min under impact velocities of 15 m/s, 20 m/s, and 30 m/s. The erosion rates of different materials increased with the increase in impact velocity, as reported by Modi et al. [32]. During the erosion process of solid particles, the particles flowing out of the sand storage tank initially had a velocity of zero. An air compressor provided pressurized air to accelerate solid particles so that the sand-carrying fluid was ultimately ejected from the nozzle at a constant impact velocity to hit the target material. In this erosion process, particles acquire kinetic energy from pressurized air. When the target material was impacted, the friction and collisions between particles and material molecules caused the material to deform, thus altering the relative positions between atoms, rearranging or breaking chemical bonds, and promoting the detachment of the material. Therefore, in order to increase the impact velocity of solid particles, it was necessary to increase the inlet air pressure. An increase in the kinetic energy gained by the particles was from the pressurized air. In this way, the material erosion was enhanced, thus leading to an increase in the erosion rate.

Under an impact velocity of 30 m/s, the maximum erosion rates of L360N, coating, and ceramic were, respectively, 0.0350 mg/g, 0.0217 mg/g, and 0.0108 mg/g. The erosion rate of L360N was approximately 1.6 times higher than that of coating and 3.2 times higher than those of ceramic. Under the same impact velocity, the erosion rates of both ceramic and coating were significantly lower than that of L360N. The difference was ascribed to the differences in material properties. The erosion rate was greatly influenced by the hardness of materials. Materials with a higher hardness exhibited a stronger local resistance to the indentation of hard objects on their surfaces. Bhosale et al. [24] also reported that the high hardness of tungsten carbide-based coatings makes their erosion resistance superior to that of the steel substrate. Therefore, with the lowest hardness, L360N had the worst resistance to solid particle erosion, thus resulting in the largest mass loss caused by solid particles. In comparison, the ceramic had the highest hardness and demonstrated the highest erosion resistance.

### 3.4. 3D Surface Profiles

**Figure 6:**
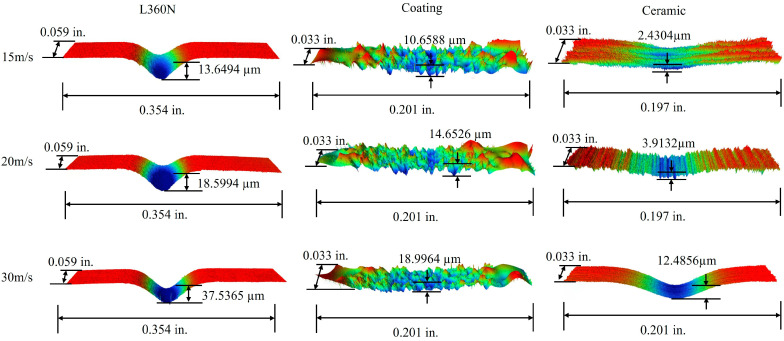
Erosion morphologies of L360N, ceramic, and coating under a confocal microscope.

**Figure 7:**
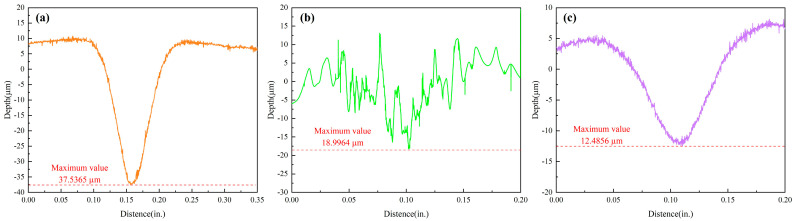
Section outlines of the three materials under an impact speed of 30 m/s: (**a**) L360N, (**b**) coating, and (**c**) ceramic.

In order to further observe the micro-morphology of the erosion area and accurately measure the erosion depth of specimens, a laser confocal microscope was employed to observe the erosion trajectories of L360N, coatings, and ceramics. The three-dimensional profiles of the eroded surfaces of L360N, coatings, and ceramics after erosion are shown in Figure 6. In the central region of erosion pits, the three-dimensional profile morphologies of eroded surfaces were completely different among the three materials, and peaks and valleys were formed on material surfaces due to the different movement directions of solid particles during the erosion process. In particular, the coating surface exhibited obvious impact traces caused by small particles, thus resulting in an uneven erosion morphology with deeper erosion depths in local areas and uneven distributed erosion pits. Due to the high hardness of the ceramic, the impact of the particles on the specimen was relatively small and stripe-shaped processing traces could be observed on the ceramic surface under the low impact velocity. The cross-sectional profiles of the specimen were obtained along the surface-erosion pit-surface in the erosion damage area (Figure 7). The cross-sectional profiles of L360N and ceramic were V-shaped and the maximum erosion depths of L360N and ceramics were 37.5365 µm and 12.4856 µm in the central region, respectively. The erosion pits on the coating surface were more than those on the L360N surface, and maximum erosion depth on coating surface was 18.9964 µm.

The erosion depths of the specimens under different impact velocities are shown in Table 6. With the increase in particle impact velocity, the impact energy also gradually increased. Solid particles exhibited higher velocity and kinetic energy under an impact velocity of 30 m/s, thus resulting in deeper and larger erosion pits compared to those under an impact velocity of 15 m/s. The result of the erosion depth was consistent with the erosion rate pattern.

To more accurately compare the erosion conditions of specimens under different impact velocities, the local erosion rates were calculated with erosion depths (Equation (3)). The maximum depth measured under a confocal microscope was determined as the erosion depth.

(3) $$E_{D}=h/t$$

where *E_D_* indicates local erosion rate (mm/d); *h* indicates erosion depth (mm); and *t* indicates erosion time (days).

**Figure 8:**
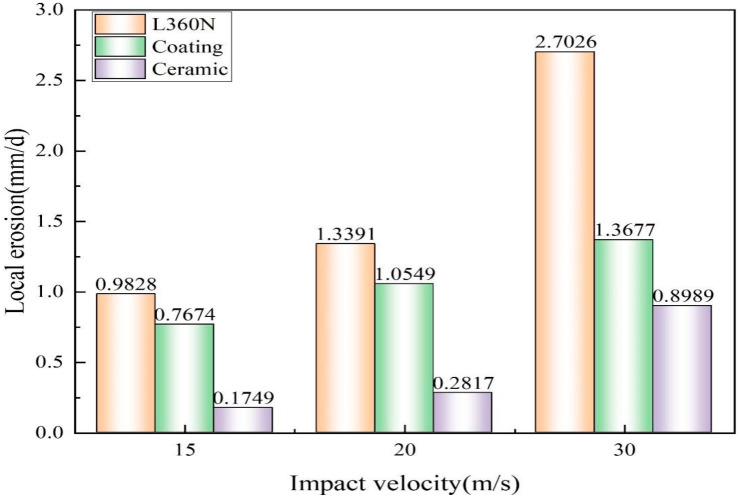
Local erosion rates of L360N, coating, and ceramic under different impact velocities.

The local erosion rate gradually increased with the increase in impact velocity (Figure 8). Under the impact velocity of 30 m/s, local erosion rates of L360N, coating, and ceramic were 2.7026 mm/d, 1.3677 mm/d, and 0.8989 mm/d, respectively. In the shale gas production process, some parts, such as elbows and tee joints, were significantly affected by local erosion. In the high sand production stage of the drainage period, the thickness of the plug used in the shale gas gathering and transportation system decreased by 43.73 mm in 20 days. In Changning Shale Gas Field, the annual erosion thickness of the desander exceeded 100 μm under a flow rate of 12 m/s. Therefore, it is necessary to take corresponding protective measures to mitigate the erosion. When the impact velocity was slower than 20 m/s, the growth trend of the local erosion rate of ceramic was relatively gentle, and the local erosion rate of the coatings was about 3.7 times that of ceramic. When the impact velocity exceeded 20 m/s, the growth trend of the local erosion rate of ceramic increased sharply, and the growth trend of coating was less significant than that of ceramic. However, the local erosion rate of the coatings still exceeded that of ceramic. Under different impact velocities, the erosion resistance of high-hardness ceramic was better than that of coating. The erosion resistance of the three materials is ranked in the following order: ceramic > coating > L360N.

### 3.5. Erosion Mechanism Analysis

**Figure 9:**
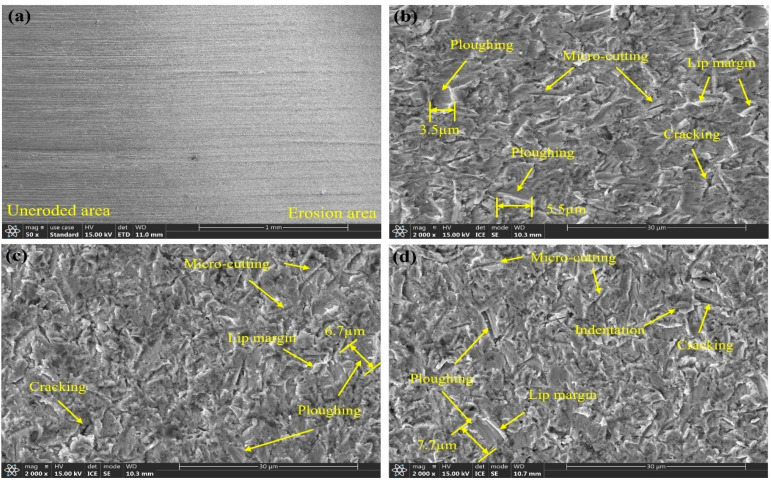
Micromorphologies of L360N under different impact velocities: (**a**) before and after erosion, (**b**) 15 m/s, (**c**) 20 m/s, and (**d**) 30 m/s.

**Figure 10:**
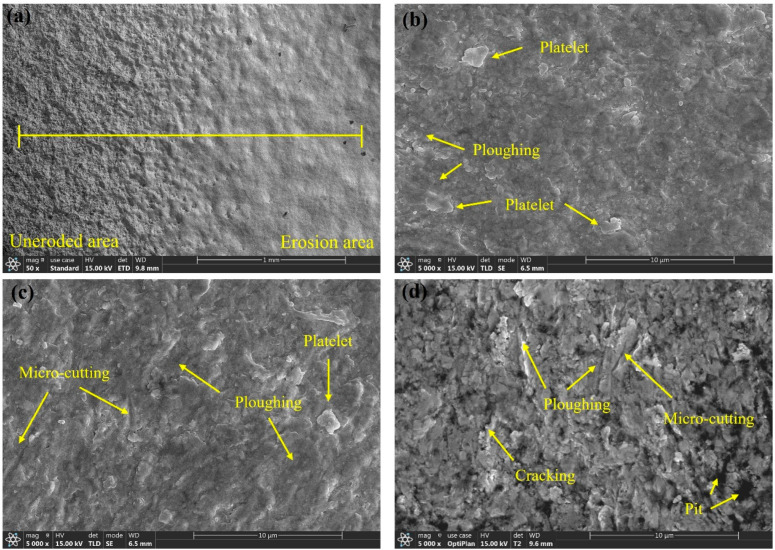
Microstructure of the coating under different impact velocities: (**a**) before and after erosion, (**b**) 15 m/s, (**c**) 20 m/s, and (**d**) 30 m/s.

**Figure 11:**
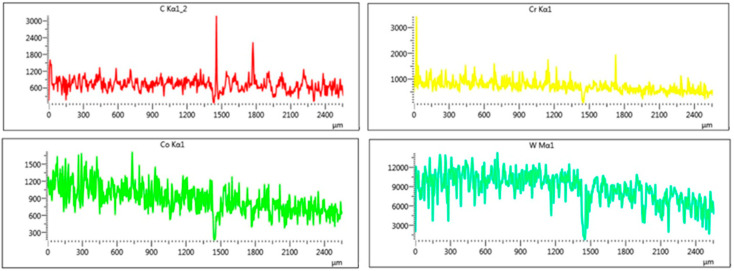
Elemental line scan result of the plating coating.

**Figure 12:**
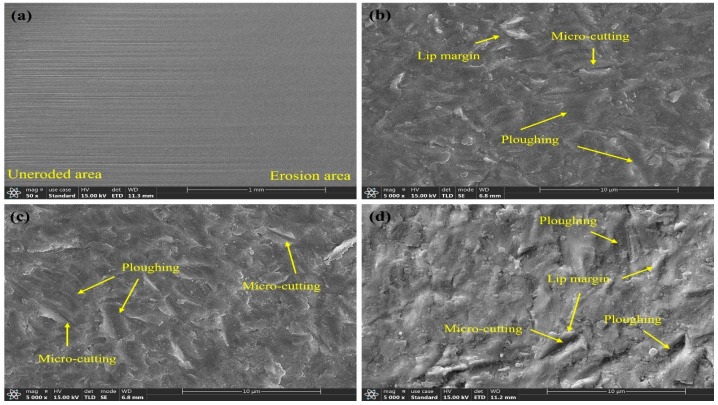
Micromorphology of ceramic under different impact velocities: (**a**) before and after erosion, (**b**) 15 m/s, (**c**) 20 m/s, and (**d**) 30 m/s.

Erosion is an extremely complex physical process. To investigate the erosion mechanisms of L360N, ceramic, and coating under different impact velocities, the microscopic morphology of the specimen surfaces was analyzed. The microscopic morphologies are shown in Figure 9, Figure 10, Figure 11 and Figure 12.

The microstructures of L360N under different impact velocities are shown in Figure 9. The microstructures of L360N before and after erosion were compared. A large number of metal processing scratches were observed on the surface of the uneroded area. However, in the eroded area, the number of scratches significantly decreased, and an obvious trace of erosion transition was observed. The main mechanisms for material removal included plowing and micro-cutting as well as indentation (Figure 9b–d). The surface of L360N exhibited wavy folds with a large number of overlapping and intersecting grooves, whose directions were consistent with the flow direction of the sand-carrying fluid. With the increase in impact velocity, the depth of plowing grooves increased from 3.5 µm to 7.7 µm, and obvious protruding lips were observed on both sides and ends of the grooves. Their formation was mainly ascribed to the plastic deformation of materials caused by scraping, micro-cutting, and plowing effects of irregular solid particles carried by high-velocity gas [8,9]. In addition, a small number of microcracks could be observed on the material surface. The continuous impact and compression of quartz sand on the material surface hardened the material and enhanced its brittleness. When the uneven stress exceeded the material threshold, cracks were generated under the impact of subsequent particles [33,34].

The force analysis of the specimens was performed under an impact angle of 30°. The specimen was subjected to a horizontal tangential force from the sand-carried fluid along its surface and a perpendicular pressure. Liu et al. [33] confirmed that the tangential force generated by erosion particles impacting the surface reached its maximum value at the lower angles between 15° and 30°. The tangential force caused by particle impact exerted a micro-cutting effect on L360N. Under this micro-cutting effect, the lips formed by the plastic deformation of the material broke into debris and detached from the surface. As solid particles further impacted the surface, new lips were formed on the surface, thus leading to the continuous mass loss of L360N [35]. The pressure generated by particle impact exerted an impact and compression effect on the materials. Repeated compression and forging by the sand-carried fluid caused the indentation depth on the material surface to further increase. Therefore, the cutting effect of the horizontal component on the specimen surface and the impact and compression effect of the vertical component led to the repeated surface plastic deformation of L360N.

The microscopic morphology of the coating under different impact velocities is shown in Figure 10. The microscopic morphology of the coating before and after erosion was compared. The surface of the non-eroded area was porous, loose, and full of holes, whereas the eroded and worn area was smooth but uneven. Under an impact angle of 30°, the main mechanisms of material removal included plowing and micro-cutting, and a small amount of irregular flake residue remained on the surface (Figure 10b–d). When the impact velocity increased to 30 m/s, small cracks and a few pits appeared on the coating surface, and numerous protruding solid particles were distributed in a scattered pattern. It was speculated that the W and Co with different hardnesses were unevenly distributed on the coating surface, resulting in a difference in the degrees of erosion and wear during the erosion process and the uneven coating surface. To investigate the elemental distribution on the coating surface before and after erosion, an elemental line scan was performed on the eroded area according to the scanning direction from the matrix to the erosion pit. The scanning line is shown in Figure 10a. The results of the elemental line scan are shown in Figure 11. The main elements on the coating surface were C, Cr, Co, and W. As the erosion depth gradually increased, the contents of Cr, Co, and W also gradually decreased and abruptly dropped at the depth of 1460 µm.

At a 30° impact angle, the cutting effect of the horizontal component on the sample surface is greater than the impact and compression effects on the vertical component. Quartz sand with small size and sharp edges causes plastic deformation of the coating due to its cutting action, and the protruding lips form flakes and peel off under repeated impacts from the quartz sand (Figure 10b). As the erosion time increases, the softer matrix within the coating peels off under the impact of quartz sand, while harder particles gradually become exposed and unevenly distributed on the coating surface, resulting in an uneven, hilly erosion morphology (Figure 6) [36]. At low angles, quartz sand with high impact energy and hardness continues to laterally impact and cut the protruding hard particles, leading to cracking and peeling of the hard particles in some locations. Additionally, a small number of microcracks can be observed on the material surface. The presence of hard particles hinders the plastic deformation of the coating, and mutual compression between them easily forms cracks at the boundaries, facilitating the peeling of the coating [37]. Since the hardness of the coating is greater than that of quartz sand, at a 30° low impact angle, the peeling of the matrix caused by micro-cutting from quartz sand is the primary reason for the erosion wear of the coating. Cracking and displacement of hard particles reduces the erosion resistance of the coating, intensifying the cutting effect of solid particles on the coating and further increasing the mass loss of the material [20].

The microscopic morphologies of ceramic under different impact velocities are shown in Figure 12. The microscopic surface morphologies of ceramic before and after erosion were compared. On the surface of the non-eroded area, a large number of machining scratches were observed, but the eroded and worn area had a smooth and flat surface. The ceramic surface was relatively smooth and flat. Only a small amount of plowing and micro-cutting traces were distributed, and no cracks or pits were observed (Figure 12b–d). Under an impact angle of 30°, the mechanisms of material removal under different impact velocities were not different and included both plowing and micro-cutting. When the impact velocity was 15 m/s, the surface micro-erosions on the ceramic surface was relatively small, and only a few protruding lips were observed. Under slow flow velocities, the horizontal component of kinetic energy was small, and the hardness of the ceramic exceeded that of quartz sand, so the cutting effect of the fluid on the ceramic was relatively weak. Under an impact speed of 30 m/s, the surface micro-erosions on the ceramic surface were significantly enhanced. The cutting effect of the fluid on ceramic under a fast flow velocity led to deeper furrows on the material surface, and many thicker lips were observed. Meanwhile, a small amount of particle detachment can be observed on the material surface. This is because at an impact velocity of 30 m/s, the bonding matrix between particles is eroded away, leading to particle detachment. However, under these experimental conditions, the ceramic surface remains firmly bonded, with no large-scale detachment observed, indicating that the dense, sintered particles of the ceramic ensure better resistance to sand erosion. It is generally believed that the erosion rate of brittle materials is the highest under an impact angle of 90°, so ceramic has the highest erosion resistance under an impact angle of 30° [38,39].

In summary, the erosion mechanisms of the three materials under different flow velocities are mainly micro-cutting and plowing. The surface of L360N showed cracking and indentation. The detachment of matrix and hard particles occurs on the coating surface. The ceramic surface remained intact without cracks or pits. Under an impact angle of 30°, for the same material, even though the microscopic morphologies formed under different impact velocities showed significant differences, the mechanisms of material removal were almost the same, indicating that the changes in particle impact velocity did not affect the erosion mechanism. As the particle impact velocity increased, the length of furrows also increased, suggesting that an increase in impact velocity enhanced the impact energy and surface tangential force of solid particles. Therefore, micro-cutting and plowing caused more severe erosion and abrasion, and the erosion rate increased. L360N exhibited the obvious characteristics of plastic material erosion and coating, and ceramic showed the erosion behavior of brittle materials. Compared with L360N, coating and ceramic had a larger hardness, better erosion resistance, and significantly higher resistance to plastic deformation under high impact velocities than L360N.

**Figure 13:**
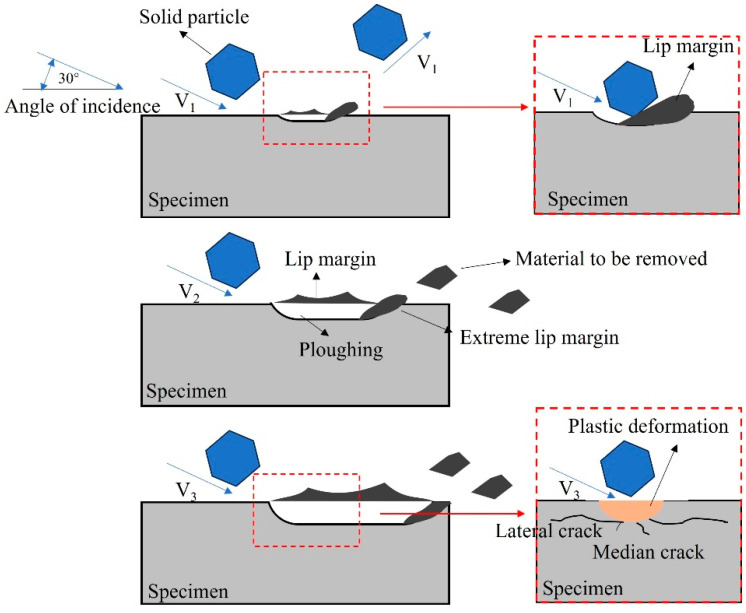
Schematic diagram of erosion mechanism under different impact velocities.

Figure 13 shows the influence of particle impact velocity on the erosion of three materials. The impact velocities of solid particles increase from top to bottom, i.e., v_1_ > v_2_ > v_3_. When particle impact velocity is v_1_, the kinetic energy acquired by solid particles from the sand-carrying fluid is relatively small. When solid particles strike the material at an impact angle of 30°, the horizontal component of kinetic energy causes plastic deformation of the material surface, leaving furrows along the cutting trajectory and extruding the material towards both sides and the front to form lips. Solid particles acquire less energy, so their energy is consumed after they travel a short distance on the material surface. Then, the cutting effect stops, and shorter furrows are formed on the material surface. Additionally, due to the slower velocity, the impact of the sand-carrying fluid on lips is relatively weak, and the lips formed on the material surface are not easily eroded. Therefore, after continuous erosion, intersecting furrows and a large number of uneroded lips are eventually left on the material surface. When the particle impact velocity is v_2_, the energy acquired by a single solid particle also increases. When a solid particle impacts the material surface at the same incident angle, the micro-cutting effect is stronger, and long and deep furrows are formed on the material surface. When the particle impact velocity is v_3_, the overlapping and intersecting furrows are more pronounced, and the erosion effect of the sand-carrying fluid on lips is also stronger under high flow velocities. The impact of the fluid can lead to the detachment of lips and increase the mass loss of the material as the flow velocity increases. When the non-uniform stress caused by impact and compression exceeds the material threshold, cracks are formed in the material.

The experimental results showed that, under any velocity, the erosion rate, erosion depth, and local erosion rate of the high-hardness ceramic were all lower than those of the coatings. Under an impact velocity of 30 m/s, the erosion rate of the coating was 2 times that of ceramic, and its local erosion rate was 1.5 times that of ceramic. Compared with ceramics, the coatings had lower hardness and weaker resistance to micro-cutting and plowing. Under high impact velocity, pits and cracks appeared on the coating surface. The erosion resistance of ceramic was much better than that of the coating. Therefore, ceramic lining materials were used to protect the easily eroded parts, such as pipeline bends and tees. Currently, erosion-resistant components such as zirconia ceramic-lined elbows and zirconia ceramic-lined three-way pipes have been applied in shale gas surface pipelines. The service life of these elbows has increased from 5 months to 2 years without any failure, and no corrosion has been found in pipelines so far. Due to its excellent corrosion resistance and erosion resistance, ceramic has played a significant protective role in shale gas surface pipelines. Additionally, thicker bends or square bends significantly reduced erosion and wear caused by proppant flowback and thus decreased the failure rate by 93%.

## 4. Conclusions

To solve the erosion problem caused by proppant flowback during shale gas production, a high-speed gas–sand jet erosion tester was used to conduct gas–solid two-phase jet erosion experiments with L360N, coating, and ceramic under simulated working conditions. The erosion wear performance of L360N, coating, and ceramic under the impact velocities of 15 m/s, 20 m/s, and 30 m/s was analyzed. The conclusions are drawn as follows:

(1) Under an impact angle of 30°, the erosion rate, erosion depth, and local erosion rate of L360N, coating, and ceramic increased with the increase in impact velocity. Under an impact velocity of 30 m/s, maximum erosion rates of L360N, coating, and ceramic were, respectively, 0.0350 mg/g, 0.0217 mg/g, and 0.0108 mg/g. Maximum erosion depths of L360N, coating, and ceramic were, respectively, 37.5365 µm, 18.9964 µm, and 12.4856 µm. Due to their high hardness, the erosion rates of the coatings and ceramics were seldom changed with an increase in impact velocity. Among the three materials, ceramic exhibited the best erosion resistance, whereas L360N had the worst erosion resistance.
(2) The erosion mechanisms of the three materials under different flow rates were primarily micro-cutting and plowing. Cracks and indentations appeared on the surface of L360N. The detachment of matrix and hard particles occurs on the coating surface. The ceramic surface remained intact, and no cracks or pits were observed. For the same material, erosion morphology varied with particle impact velocity, but the change in particle impact velocity did not affect the primary erosion mechanism. Increasing the impact velocity increases the impact energy and tangential force of solid particles on the surface, thereby increasing the erosion rate.
(3) The erosion rate, erosion depth, and local erosion rate of high-hardness ceramics were all smaller than those of coating. The erosion resistance of ceramics was much better than that of coatings. Therefore, erosion-resistant components such as zirconia ceramic-lined bends and zirconia ceramic-lined tees were used to protect shale gas surface pipelines. After two years of service, no failure occurred, and the erosion and wear problem of surface pipelines caused by proppant flowback was solved.

## Figures and Tables

**Table 1:** Chemical composition of L360N (wt%).

Material	Chemical Composition										
	C	Ni	Cr	Mn	Si	P	S	V	Mo	Cu	Fe
L360N	0.15	0.019	0.04	1.350	0.380	0.011	0.005	0.003	<0.004	0.013	Bal.

**Table 2:** Chemical composition of ceramics (wt%).

Material	Chemical Composition			
	O	Y	H_f_	Zr
Ceramic	13.97	3.24	1.52	Bal.

**Table 3:** Chemical composition of coatings (wt%).

Material	Chemical Composition				
	C	O	Cr	Co	W
Coating	6.09	1.66	3.63	11.91	Bal.

**Table 4:** Experimental scheme of jet erosion under a gas–solid two-phase flow.

Experimental Parameters	Setting
Erodent	Quartz sand
materials	L360N, ceramic, and coating
Impact velocity (m/s)	15, 20, and 30
Experimental temperature (°C)	Room temperature
Impact angle (°)	30
Particle mass flow (g/min)	2
Test duration (min)	20

**Table 5:** Vickers hardness of three materials.

Materials	Vickers Hardness (HV)					
	Test 1	Test 2	Test 3	Test 4	Test 5	Means
L360N	221	226	202	268	240	231
Ceramic	1749	1703	1649	1469	1652	1645
Coating	1072	1065	1122	1063	1036	1072

**Table 6:** Erosion depth of specimens under different impact velocities.

Materials	L360N	Coating	Ceramic
Impact Velocity (m/s)	Maximum Erosion Depths (µm)		
15	13.6494	10.6588	2.4304
20	18.5994	14.6526	3.9132
30	37.5365	18.9964	12.4856

## Data Availability

The data presented in this study are available on request from the corresponding author due to privacy restrictions.

## Supplementary Materials

The following supporting information can be downloaded at: https://www.mdpi.com/article/10.3390/ma17174278/s1, Figure S1. Particle size distribution of quartz sand. Figure S2. Microstructure of quartz sand particles.
