# Supplementary material for: Research on the Erosion Law and Protective Measures of L360N Steel for Surface Pipelines Used in Shale Gas Extraction
Source: Materials (Basel). 2024 Aug 29;17(17):4278. doi: 10.3390/ma17174278 (PMC11395843; doi:10.3390/ma17174278)
Supplement: Supplementary file 1 [file materials-17-04278-s001.zip › materials-3116675-supplementary.pdf]

Supplementary Material

Erosion Mechanism of the Gas–Solid Two-Phase Flow in Surface Pipelines for Shale Gas in the Sichuan Basin

Shaoquan Huo [1], Lincai Peng [1,*], Yunpeng Li [2], Yong Xu [3], Hongbing Huang [1], Xi Yuan [1]

[1] Research Institute of Natural Gas Technology, PetroChina Southwest Oil and Gasfield Company, Chengdu 610213, China

[2] Chengdu Natural Gas Chemical Plant, PetroChina Southwest Oil and GasField Company, Chengdu 610213, China

[3] PetroChina Southwest Oil and Gasfield Company, Chengdu 610051, China

*Correspondence: lincai.peng@foxmail.com

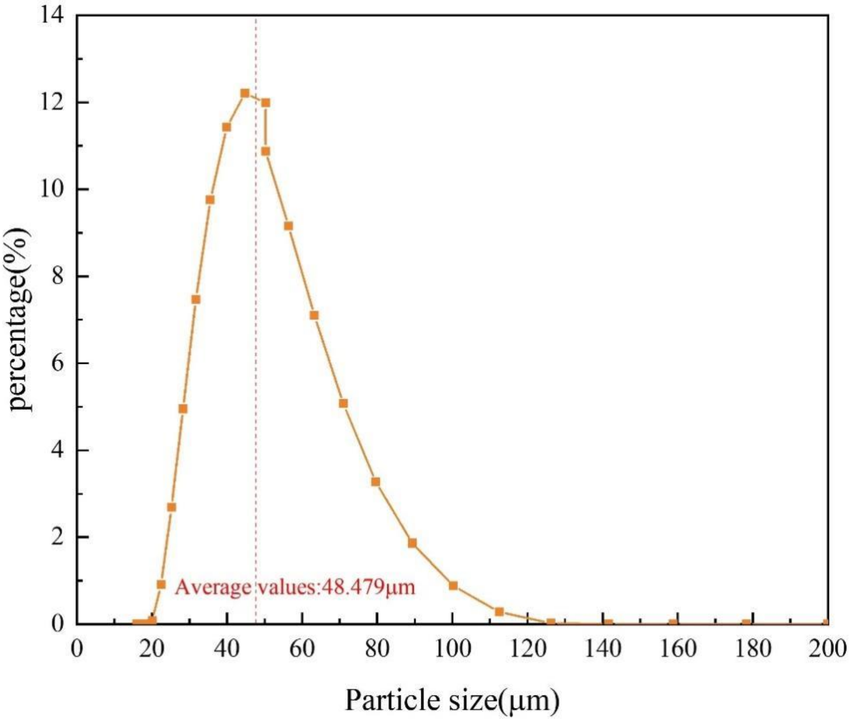

Figure S1 Particle size distribution of quartz sand.

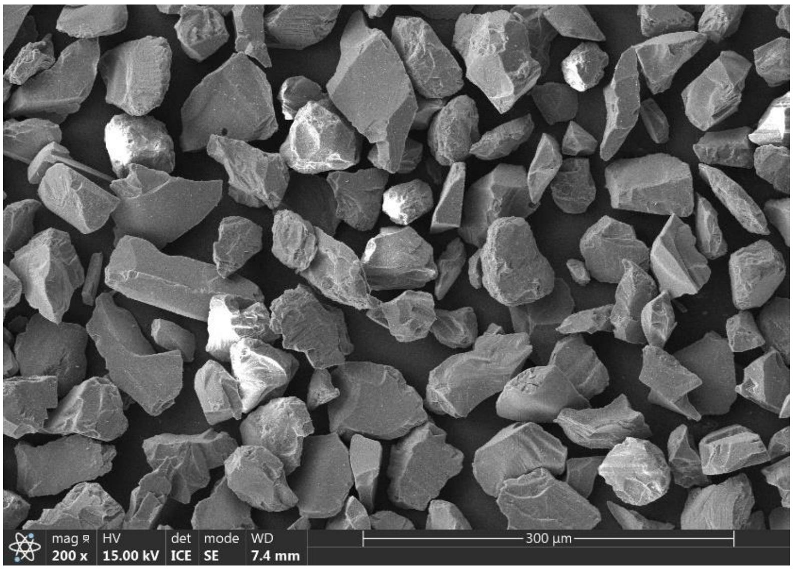

Figure S2 Microstructure of quartz sand particles.
